# Prime Time for HLA Desensitization: Imlifidase in the Spotlight

**DOI:** 10.3389/ti.2023.11616

**Published:** 2023-06-28

**Authors:** Oriol Bestard, Francesc Moreso, Anthony Dorling

**Affiliations:** ^1^ Kidney Transplant Unit, Nephrology Department, Vall d’Hebron University Hospital, Barcelona, Spain; ^2^ Centre for Nephrology, Urology and Transplantation, King’s College London, London, United Kingdom

**Keywords:** transplant access, desensitization, rejection, survival benefit, HLA allosensitization

An increasing number of highly human leukocyte antigen (HLA) sensitized patients are currently on kidney transplant waiting lists worldwide. The leading causes of sensitization are previously failed transplants, previous pregnancies, or blood transfusions. Because the HLAs to which patients have been previously exposed—or to which they have made an anti-HLA antibody—are listed with organ allocation bodies as “unacceptable,” sensitized patients have a significantly reduced chance of finding an HLA compatible donor. Thus, they can wait for a very long time on chronic dialysis therapy, which has deleterious consequences in terms of mortality, quality of life, and healthcare costs.

In the last decade, different strategies have been developed and implemented in many countries, aimed at increasing the likelihood of finding HLA compatible organs for these patients, whilst maintaining a balanced equity access to kidney transplantation for all waitlisted patients. These include sliding-scale prioritization score programs, the acceptable mismatch program, and the expansion of kidney-pair exchange programs [1]. While all these strategies have been variously successful for many highly sensitized patients, a group of (very) highly sensitized individuals (>99.9% cPRA) have failed to benefit, and this expanding group still have an extremely low chance of finding a HLA compatible organ.

There are two approaches to improve the chances of these patients; “de-listing” which involves ignoring selected low-level HLA antibodies in the allocation process, and “desensitization” to reduce higher levels of HLA antibodies down to permissive levels that can then be ignored. These approaches offer no survival disadvantage [2] to waiting for a well-matched organ but are associated with higher rates of early and late aggressive rejection and a shortened graft half-life. Unfortunately, many patients have no delisting options, and current desensitization strategies have failed to demonstrate a consistent success, especially for those without a living donor. Therefore, there is an important unmet need for new drug development enabling access to transplantation to these (very) highly sensitized kidney transplant candidates [3].

Notably, a new drug Imlifidase, a recombinant cysteine protease with an unprecedented capacity to rapidly cleave all four IgG subclasses, both soluble and on the B-cell surface [4], has the potential to offer hope for these patients. By transiently depleting all circulating IgG, including HLA antibodies for several days, Imlifidase can uniquely create a window of opportunity for highly sensitized patients with high pathogenic anti-HLA antibody levels to undergo kidney transplantation [5]. Two phase I/II clinical trials [6] have demonstrated the capacity of Imlifidase to effectively convert a positive crossmatch to a negative one, leading to optimal 3- year graft and patient survival rates. Even though high rates of antibody-mediated rejection (ABMR) were observed in these studies, occurrence of hyperacute or accelerated rejection was avoided. Based on these data, the EMEA recently provided a rapid marketing authorization approval throughout the European Union, conditioned to the outcomes of three on-going studies (17-HMedIdeS-14, 20-HMedIdeS-19, 17-HMedIdeS-20) and thus, it has become the first immunosuppressant approved for HLA desensitization in highly sensitized kidney transplant candidates of deceased donors.

Although an outstanding achievement, the use of Imlifidase has major implications for organ allocation systems, alters current algorithms for immune-risk stratification, and impacts on immunosuppression management. Most of these issues are directly related to the pharmacokinetic/pharmacodynamic nature of Imlifidase [4], which needs to be thoroughly understood; the transient effect of Imlifidase leads to a progressive IgG antibody repopulation in 3–5 days, the immunogenicity of the drug precludes repeated doses and importantly, the broad IgG cleavage effect also targets any (IgG) antibody-based therapy, thus precluding the use of most frequent induction therapies used in these patients. Furthermore, for patients to receive an Imlifidase-enabled deceased kidney organ offer, a thorough immunological evaluation must be conducted, including both an anti-HLA antibody de-listing strategy to reduce the virtual cPRA burden and also establish what is an acceptable positive cross-match (XM) (virtual and/or cell-based) to ultimately decide whether to undergo or abort kidney transplantation.

In this issue of *Transplant International* [7], a French expert transplant group endorsed by different French scientific societies (SFT, FNDT, SFHI), propose a set of clinical, immunological, and therapeutic recommendations on how to implement Imlifidase in clinical transplantation. The authors should be acknowledged for the thorough description of the different recommendations provided in this consensus report, especially considering the relatively low level of evidence currently available in this topic. Even though some recommendations are based on their national allocation policy, most of them may be perfectly generalized to any other transplant system worldwide.

Assessing transplant candidates eligible for Imlifidase is considered in four main areas. First, the authors highlight the importance of selecting only those highly sensitized candidates who have extremely low chances of finding a HLA compatible transplant, according to the distinct national prioritization programs available (Table 1); in France this threshold is established by having a persistent cPRA ≥ 98% with a waitlist time of at least 3 years. While these thresholds may be relatively arbitrary, an objective calculation of the cPRA burden and time to receive an organ offer should be country/region-specific to maintain a transparent balance of access to transplantation between highly sensitized and non-sensitized transplant candidates. Sensitized patients with an urgent transplant need because of lack of vascular access could eventually be considered. Authors limit the use of Imlifidase to patients with no more than two previous transplants. While this may be understandable due to the scarcity of organs, the time onset of end-stage kidney disease should be considered, as this exclusion criteria may significantly hamper access to transplantation to pediatric patients who have a longer lifespan and thus, may need more than two transplants. Secondly, they recommend careful consideration of relevant recipient and donor characteristics: Avoiding recipients at higher risk of life-threatening opportunistic infections or cardiovascular-related events may minimize fatal outcomes and limit organ offers to those without severe acute kidney injury or long cold ischemia times (CIT), to help reduce the risk of delayed graft function and organ immunogenicity while maximizing the capacity of the organ to overcome allogenic insults. Thirdly, the de-listing strategy recommended by authors is designed to minimize the risk of highly pathogenic antibody rebound after transplantation. They suggest avoiding de-listing antibodies with MFI values above those which highly correlate with both complement-fixing abilities, a strategy which may be further refined by using C1q or C3d assays. In addition, those antibodies were significantly reduced after a 1:10 dilution (<5000 MFI), and are also recommended for a first delisting. Importantly, since incompatible (donor specific antibody (DSA) positive) kidney transplantation with negative cell-based XM (both FCM and CDC-XM) may be feasible without Imlifidase [2], the authors describe a plausible MFI threshold (>6000 MFI) to infer the presence of a positive FCM but negative CDC-XM to accept with the use of Imlifidase. While this approach has high inter-laboratory variability due to the different type of SAFB used, it may simplify the logistics for those transplant programs not routinely performing cell-based XM, and ultimately reduce CIT. Notably, delisting antibodies may be performed in a stepwise approach, starting from less to more aggressive antibodies according to the reduction of the cPRA burden and thus, the likelihood of receiving a transplant offer. Finally, they consider immunosuppression management, and recommend pre-delisting rituximab, followed by T-cell depletion, high-dose IVIG, and rituximab after day 4 post-transplantation, in the context of tacrolimus, mycophenolate mofetil, and steroids beginning on day 0.

**TABLE 1:** Main immunological, demographic and clinical variables influencing desicion-making regarding the use of Imlifidase for desensitization.

	Immunological characteristics	Clinical characteristics
**Patient selection**	**Delisting strategy**	**Recipient**
	Step-wise strategy based on recent serum MFI Ab values	Sensitized patients with urgent Tx needs
	> 6000 MFI (<5000 after 1:10 dilution)	Highly sensitized with long waiting time in proritization programs
	C1q/C3d negative	No vascular access
	Reduction of Ab titers after Serial dilutions	Avoid frail candidates
	High MFI values of Ab against repeated antigens in previous transplants^a^	Increased IS burden (induction and rejection rescue theapies high risk of AR)
		For cause/surveillance bx
		Optimal Haemodynamics (BP)^a^
**At Transplantation**	**Prior to Imlifidase**	**Donor**
	Negative CDC-XM	Minimize the risk of post-Transplant DGF
	No FC-XM with positive virtual XM:	Long CIT
	DSA MFI >6000 in sera with <5000 MFI after 1:10 dilution (C1q/C3d negative)	severe AKI
	Availability of FC-XM^a^:	Avoid (very) ECD
	Positives (pronase-treated) B/T-cell FC-XM	
	**Post-Imlifidase** ^a^	
	Negatives FC and CDC-XM	

^a^ Recommendations not made by the French expert group but suggested by the authors of this editorial comment.

In summary, the advent of Imlifidase may revolutionize the field of HLA desensitization and opens a new window of opportunity for an increasing number of patients with extremely low chances of finding an HLA compatible organ. Although there is still more to be learned from ongoing, prospective, controlled trials it is imperative that clinical and immunological data is gathered from Imlifidase programs as they begin in multiple countries and it is highly recommended that Imlifidase should only be used in a rigorous and controlled manner as suggested by the authors here. There is an ideal opportunity, with the use of Imlifidase, to better comprehend the complex mechanism of alloimmune sensitization and allow a better understanding of the dynamics and pathogenicity of the humoral immune response. Importantly, all this effort would be highly advisable to be undertaken within international, cooperative scientific networks and endorsed by national and international transplant societies.

## Data Availability

The original contributions presented in the study are included in the article/supplementary material, further inquiries can be directed to the corresponding author.

## References

[1] Mamode N Bestard O Claas F Furian L Griffin S Legendre C European Guideline for the Management of Kidney Transplant Patients with HLA Antibodies: By the European Society for Organ Transplantation Working Group. Transpl Int (2022) 35:10511. 10.3389/ti.2022.10511 36033645 PMC9399356

[2] Manook M Koeser L Ahmed Z Robb M Johnson R Shaw O Post-Listing Survival for Highly Sensitised Patients on the UK Kidney Transplant Waiting List: a Matched Cohort Analysis. Lancet (2017) 389(10070):727–34. 10.1016/S0140-6736(16)31595-1 28065559

[3] Bestard O Couzi L Crespo M Kessaris N Thaunat O. Stratifying the Humoral Risk of Candidates to a Solid Organ Transplantation: a Proposal of the ENGAGE Working Group. Transpl Int (2021) 34:1005–18. 10.1111/tri.13874 33786891

[4] Huang E Maldonado AQ Kjellman C Jordan SC. Imlifidase for the Treatment of Anti-HLA Antibody-Mediated Processes in Kidney Transplantation. Am J Transpl (2021) 22:691–7. 10.1111/ajt.16828 PMC9293130 34467625

[5] Jordan SC Lorant T Choi J Kjellman C Winstedt L Bengtsson M IgG Endopeptidase in Highly Sensitized Patients Undergoing Transplantation. N Engl J Med (2017) 377(5):442–53. 10.1056/nejmoa1612567 28767349

[6] Kjellman C Maldonado AQ Sjöholm K Lonze BE Montgomery RA Runström A Outcomes at 3 Years post-transplant in Imlifidase-Desensitized Kidney Transplant Patients. Am J Transpl (2021) 21:3907–18. 10.1111/ajt.16754 PMC9290474 34236770

[7] Couzi L Malvezzi P Amrouche L Anglicheau D Blancho G Caillard S Imlifidase for Kidney Transplantation of Highly Sensitized Patients with a Positive Crossmatch: The French Consensus Guidelines. *Transpl Int* (2023) 36:11244. 10.3389/ti.2023.11244 PMC10336835 37448448

